# Comprehensive Associations Between Spinal–Pelvic Alignment and Muscle Shortening in Healthy Young Men: An Analysis of Individual and Interactive Effects in the Sagittal Plane Using SHapley Additive exPlanation

**DOI:** 10.3390/jfmk10030259

**Published:** 2025-07-09

**Authors:** Minami Akao, Yuna Ishikura, Takuma Isshiki, Shinnosuke Tsukada, Hayato Shigetoh, Junya Miyazaki

**Affiliations:** Department of Physical Therapy, Faculty of Health Science, Kyoto Tachibana University, 34 Yamada-cho, Oyake, Yamashina-ku, Kyoto 607-8175, Japan; a903022004@st.tachibana-u.ac.jp (M.A.); a903022028@st.tachibana-u.ac.jp (Y.I.); a903022032@st.tachibana-u.ac.jp (T.I.); a903022164@st.tachibana-u.ac.jp (S.T.); j-miyazaki@tachibana-u.ac.jp (J.M.)

**Keywords:** sagittal spinal alignment, pelvic tilt, muscle shortening, SHAP (SHapley Additive exPlanation), posture analysis, machine learning

## Abstract

**Objectives**: To comprehensively examine the association between spinopelvic alignment and muscle shortening in healthy young men, focusing on the individual and interactive effects of thoracic kyphosis, lumbar lordosis, and anterior pelvic tilt using SHapley Additive exPlanation (SHAP) analysis. **Methods**: Forty-one healthy young adult men participated in this cross-sectional study. Thoracic kyphosis, lumbar lordosis, and anterior pelvic tilt were measured using a flexible curve ruler and inclinometer. Muscle length indices for six muscles (iliopsoas, rectus femoris, gluteus maximus, hamstrings, back extensors, and abdominals) were assessed via standardized physical examinations and image analysis. A machine learning model was developed, and SHAP analysis applied to determine individual and interactive contributions of spinopelvic angles to each muscle length index. **Results**: SHAP analysis showed that hip-related muscle shortening (iliopsoas, rectus femoris, hamstrings, gluteus maximus) was influenced by both individual alignments and interactions, especially between thoracic kyphosis and lumbar lordosis. Lumbar lordosis was most associated with iliopsoas shortening (SHAP = −0.09), while anterior pelvic tilt was linked to hamstring shortening (SHAP = −0.30). Thoracic kyphosis was the key factor for rectus femoris shortening (SHAP = −0.05). Interactive effects exceeded individual contributions for the rectus femoris, gluteus maximus, and hamstrings. In contrast, spinal alignment had minimal influence on the back extensors and abdominals. **Conclusions**: Both individual and intersegmental spinal alignments are associated with muscle shortening, particularly in hip-related muscles. The interaction between thoracic kyphosis and lumbar lordosis plays a pivotal role. These findings underscore the importance of evaluating segmental spinal interactions when assessing muscle flexibility and posture.

## 1. Introduction

Postural alignment in humans is one of the critical elements influencing the activities of daily living and physical function. Standing posture is the state wherein the center of the body mass is maintained within the base of support and the body is aligned vertically [1]. In particular, the sagittal-plane standing posture is affected by the spinal alignment curvature, including thoracic kyphosis and lumbar lordosis, and is associated with displacement of the center of mass. One of the representative classifications based on the sagittal spinal curvature is the Kendall classification, which categorizes posture into four types based on the characteristics of the spinal curvature: ideal posture, kyphotic–lordotic, flat back, and sway back [2]. Abnormal sagittal spinal curvature is reportedly associated with deviations in postural alignment and adverse health outcomes, including pain, reduced mobility, impaired physical function, and increased mortality [3]. The spinal sagittal curvature is not limited to isolated regions such as the thoracic or lumbar spine. However, it is regulated by the spinal–pelvic complex, which reflects its interrelationships with other body regions. Therefore, to understand normative alignment in the sagittal plane, it is essential not only to evaluate spinal curvature alone but also to analyze each postural alignment type by considering the positional relationship of the spinal-pelvic complex in relation to other body regions [4].

Muscle shortening is one factor associated with the spinal alignment. The shortening or overactivity of specific muscle groups may be related to trunk inclination [3]. Janda classified muscles into two groups: tonic muscles, which contain a higher proportion of red fibers and are responsible for sustained postural contractions; and phasic muscles, which have a higher proportion of white fibers and are involved in rapid, dynamic joint movements [5,6]. Tonic muscles are prone to tightness or shortening, whereas phasic muscles are more susceptible to weakness. Postural alignment during standing is thought to be associated with muscle length. For example, the shortening of the hip flexors is associated with anterior pelvic tilt, whereas the shortening of the hip extensors is associated with posterior pelvic tilt [2]. When the lumbar erector spinae are shortened and the abdominal muscles are relatively lengthened, the pelvic tilt and lumbar lordosis tend to increase beyond the normal ranges. This suggests that pelvic tilt is related to lumbar curvature in normal standing posture and that both are influenced by the performance and length of the trunk muscles [7]. Thus, muscle shortening is likely associated with the alignment of the pelvis and lumbar spine. However, conflicting findings have been reported, with some studies indicating that hamstring muscle length is not associated with pelvic tilt angle [8]. Walker et al. suggested that abdominal muscle length may also be associated with lumbar lordosis and pelvic tilt [9]. However, a combination of complex factors can influence this relationship. Therefore, postural alignment may not be determined solely by muscle shortening. In summary, while some studies report associations between spinal alignment and muscle shortening, others report no such relationship; the association remains inconclusive.

Although previous studies have suggested a potential relationship between individual spinal alignment and muscle shortening, spinal alignment is inherently interrelated across different spinal regions. Therefore, interactions between spinal regions may also contribute to muscle shortening. However, the relationship between individual spinal alignments and interregional interactions related to muscle length has not been sufficiently explored. Recent studies have begun to apply machine learning approaches to elucidate complex biomechanical interactions within the spinal–pelvic region. For example, Pasha et al. demonstrated the utility of machine learning analysis in interpreting spinal alignment features from spinal imaging data, highlighting the potential of explainable AI in musculoskeletal research contexts [10]. However, few studies have extended such approaches to analyze the contribution of spinal alignment patterns to muscle length indices, particularly in a standing posture. Clarifying the relationship between spinal alignment and muscle length could enhance clinical decision-making for assessment and treatment. These insights may support the development of individualized interventions to improve postural alignment, flexibility, and overall physical performance. Accordingly, the present study aimed to investigate the association among muscle length, individual contributions, and interregional interactions of spinal alignment, focusing specifically on the thoracic, lumbar, and pelvic regions.

## 2. Materials and Methods

### 2.1. Participants

The participants were 41 healthy young adult men (mean age: 20.7 ± 0.6 years; height: 170.5 ± 6.0 cm; weight: 61.9 ± 9.8 kg; BMI: 21.2 ± 2.7). The exclusion criteria included a history of spinal fractures or other spinal disorders, known neurological conditions, and spinal asymmetry. Specifically, participants with a known diagnosis of scoliosis made by a medical professional were excluded, as radiographic assessment (e.g., Cobb angle) was not performed in this study. This study was approved by the ethics committee of Kyoto Tachibana University (approval no. 24-74). All participants received a verbal explanation of the study and provided written informed consent before participation.

### 2.2. Study Protocol

All participants underwent the following measurements: muscle length indices, spinal curvature angles, and pelvic tilt angles. All measurements were conducted under standardized conditions, with the participants wearing swimwear for the lower body and no clothing for the upper body.

### 2.3. Measurement of Spinal Curvature Angles

**Figure 1:**
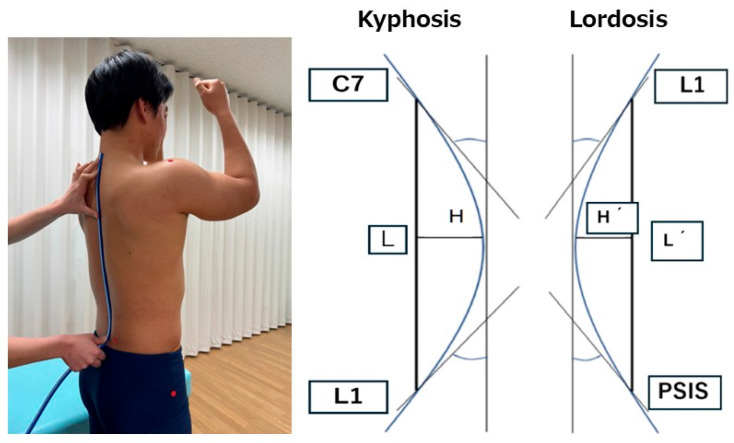
The evaluation of spinal alignment using a flexible curve ruler and method for calculating spinal curvature angles.

Spinal curvature angles were measured in a static standing posture using a flexible curve ruler (Shinwa Rules Co., Ltd., Niigata, Japan). The participants stood barefoot with their feet shoulder-width apart and maintained a static standing posture while flexing their shoulder and elbow joints to 90° [11] (Figure 1). Anatomical landmarks were identified, and markers were placed on the spinous processes of the seventh cervical vertebra (C7) and the first lumbar vertebra (L1), as well as on the left and right posterior superior iliac spines (PSIS). The flexible curved ruler was molded along the contour of the spine by gently pressing it with the fingers to ensure that it conformed closely to the spinal shape. The ruler was then carefully transferred to the paper without deformation and traced [12]. This procedure was repeated twice, and the mean value was used for the analysis. Following the method described in previous studies [12], the thoracic kyphosis angle was calculated using a straight line (L) drawn between C7 and L1 and a perpendicular line (H) from the apex of the curve to the L.

Thoracic kyphosis angle = 4 × arctangent (2H/L)

Similarly, the lumbar lordosis angle was calculated using a straight line (L′) drawn between L1 and the midpoint of the left and right PSIS and a perpendicular line (H′) from the apex of the curve to L′ (Figure 1).

Lumbar lordosis angle = 4 × arctangent (2H′/L′)

### 2.4. Measurement of Sagittal Pelvic Alignment

Sagittal pelvic alignment was measured using an inclinometer (BWT61 Gyroscope Sensor; WitMotion Co., Ltd., Shenzhen, China). The measurement posture was identical to that of the spinal curvature assessment: participants stood barefoot with feet shoulder-width apart, maintaining a static standing posture with both the shoulder and elbow joints flexed to 90°. The inclinometer was placed at the midpoint between the left and right posterior superior iliac spines (PSIS), and the angle relative to the vertical axis was measured. The anterior tilt angle of the sacrum, which is related to the pelvic tilt, was used to indicate the anterior pelvic inclination [13].

### 2.5. Measurement of Muscle Length Indices

To comprehensively assess the flexibility of the primary hip flexor and extensor muscle groups as well as the trunk muscles, muscle length indices were measured for six specific muscles: the iliopsoas, rectus femoris, gluteus maximus, hamstrings, abdominal muscles, and back extensor muscles. Lateral images of the participants were captured using the rear camera of a 7th-generation iPad (Apple Inc., Cupertino, CA, USA) running iPadOS 16.3.1. The device was positioned approximately 3 m from the participant at the level of the greater trochanter to ensure full-body visibility in the frame. Images were taken using the default camera application, and the resolution was 1920 × 1080 pixels, consistent with the standard output of the device. Angle measurements were performed using ImageJ image analysis software (NIH, Bethesda, MD, USA; available from https://imagej.net/ij/download.html; accessed on 13 May 2025), with anatomical landmarks marked at the acromion, greater trochanter, lateral femoral epicondyle, and lateral malleolus.

**Figure 2:**
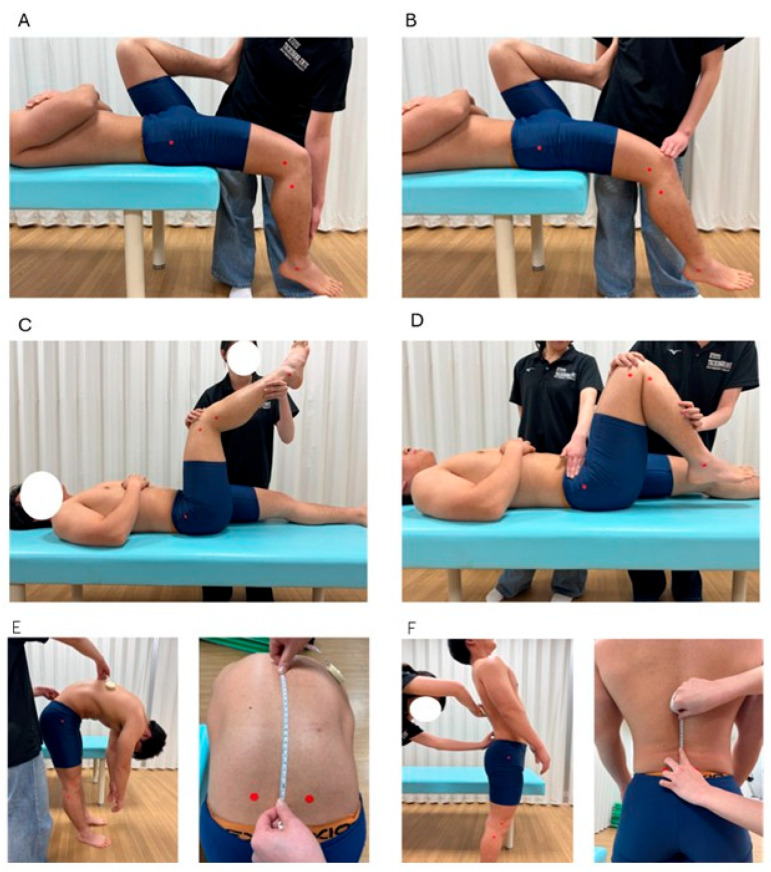
Measurement methods for each muscle length index. (**A**): rectus femoris; (**B**): iliopsoas; (**C**): hamstrings; (**D**): gluteus maximus; (**E**): back extensor muscles; (**F**): abdominal muscles.

For the rectus femoris and iliopsoas, a modified Thomas test was used to calculate the hip and knee joint angles [14]. The participants lay in a supine position with both legs hanging off the edge of the examination table. The non-measured leg was brought as close to the chest as possible while maintaining contact between the lumbar spine and table. The leg to be measured remained relaxed and hanging below the table. The reference axis for the rectus femoris was defined as the femoral shaft (the line between the greater trochanter and lateral femoral epicondyle), and the movement axis was defined as the lower leg shaft (the line between the fibular head and lateral malleolus) (Figure 2A). For the iliopsoas, the reference axis was the line between the acromion and greater trochanter, and the movement axis was the femoral shaft (Figure 2B).

The participants lay supine with their hip and knee joints flexed to 90° to evaluate the hamstrings. The knee was extended as much as possible from this position, and the knee joint angle was measured at full extension [14]. The reference axis was the femoral shaft and the movement axis was the shaft of the lower leg (Figure 2C).

For the gluteus maximus, with the anterior superior iliac spine (ASIS) stabilized, the hip joint was passively flexed while keeping the knee flexed. The hip flexion angle was measured when the ASIS began to move [15] (Figure 2D). The reference axis was the line from the acromion to the greater trochanter and the movement axis was the femoral shaft.

A line connecting the left and right PSIS was drawn to assess the back and abdominal muscles, and a point 15 cm above the intersection with the spine was used as a landmark. The participants flexed and extended their trunks while keeping their arms relaxed. The distance between the two landmarks during flexion and extension was measured using a tape measure, and the change in distance was used as the muscle length index [16] (Figure 2E,F). An increase in the distance during flexion and a decrease during extension indicate greater lumbar mobility.

The rectus femoris, iliopsoas, hamstrings, and gluteus maximus were measured on both sides, and the side showing the most significant muscle shortening was used for the analysis. All measurements were performed twice, and the minimum value was used for analysis.

### 2.6. Data Analysis

To investigate the individual and interactive effects of thoracic, lumbar, and pelvic alignment on muscle length, we first constructed random forest regression models for each muscle. The dataset comprised 41 participants, and each model used 17 spinopelvic alignment parameters as input variables to predict a single muscle length index. The models were built using scikit-learn with 100 estimators (n_estimators = 100) and a fixed random state (random_state = 42) for reproducibility. All other hyperparameters were set to default. Given the limited sample size, we did not use cross-validation but instead assessed model performance using mean absolute error (MAE) to quantify prediction accuracy. No normalization (e.g., z-score standardization or min–max scaling) was applied to the muscle length indices, as the values were already expressed in interpretable physical units (degrees or centimeters), making further transformation unnecessary. Subsequently, we employed SHAP analysis, a model interpretation method based on machine learning. SHAP is a game-theoretic approach that quantifies the contribution of each independent variable to the predicted value of the dependent variable [17]. The SHAP values indicate the magnitude and direction of each variable’s contribution to the prediction, not causality. A positive SHAP value implies that the variable increases the predicted outcome, whereas a negative value indicates a contribution that decreases the prediction.

In this study, the dependent variables were the muscle length indices, and the independent variables were the thoracic kyphosis, lumbar lordosis, and anterior pelvic tilt angles. A negative SHAP value for the muscle length index was interpreted as a tendency toward muscle shortening, except for the abdominal muscles, for which a positive SHAP value indicates shortening. To evaluate the individual effects of spinal alignment, SHAP summary plots and mean SHAP values were used to visualize and quantify the relative contribution of each angle to muscle length indices. The summary plot shows the contribution of each independent variable (SHAP value) to the prediction, providing a comprehensive view of the variables that influence muscle-shortening tendencies. Additionally, to assess the interactions between spinal regions, we calculated the mean interaction SHAP values to examine their combined influence on the muscle length indices. The interaction SHAP quantifies the contribution of the interaction between two variables to the predicted outcome. We also visualized the relationship between the SHAP values and spinal curvature angles using dependence plots, focusing on the spinal alignment variable with the most substantial contribution (individually or through interaction). In the dependence plot, the x axis represents the feature value, the y axis represents the SHAP value, and each point represents an individual participant. This allows for a visual interpretation of how feature values relate to the prediction contributions. Moreover, dependence plots can visualize the interaction effects using color-coding points based on the value of a second variable. When interaction effects are present, noticeable shifts in the color patterns are observed, indicating the presence and nature of the interaction.

## 3. Results

### 3.1. Characteristics of Participant Measures

The characteristics of the spinal alignment and muscle length indices of the study participants are presented in Table 1.

### 3.2. Effects of Spinal Alignment on Muscle Length Indices

**Figure 3:**
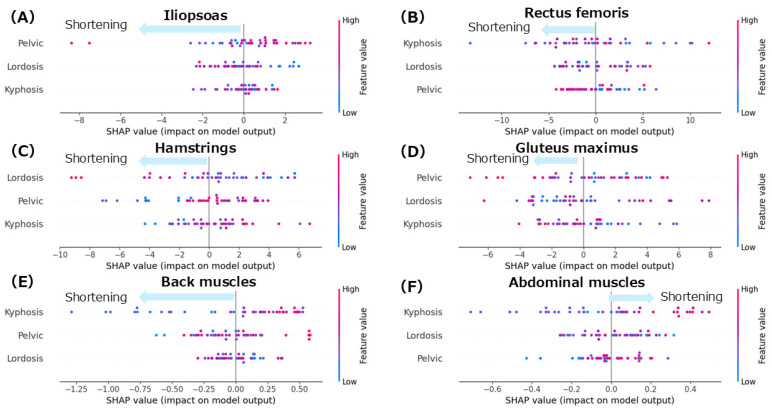
SHAP summary plots for each muscle length index. (**A**): iliopsoas; (**B**): rectus femoris; (**C**): hamstrings; (**D**): gluteus maximus; (**E**): back extensor muscles; (**F**): abdominal muscles. Color coding in the SHAP summary plots indicates the magnitude of each spinal alignment parameter: red represents higher values of the corresponding feature (e.g., greater kyphosis, lordosis, or pelvic tilt), while blue indicates lower values.

The mean absolute error (MAE) for each model was as follows: iliopsoas, 3.2°; rectus femoris, 8.1°; gluteus maximus, 8.6°; hamstrings, 7.1°; back extensor muscles, 0.9 cm; and abdominal muscles, 0.6 cm. These values indicate that the models reasonably approximated each muscle length index based on spinopelvic alignment parameters. Figure 3 shows the SHAP summary plots for each muscle length index derived from the random forest model. The features are ranked in descending order based on their mean absolute SHAP values, indicating their relative contributions to the model’s prediction. Table 2 displays the mean SHAP values for the individual and interaction effects of spinal alignment on each muscle length index.

#### 3.2.1. Iliopsoas

**Figure 4:**
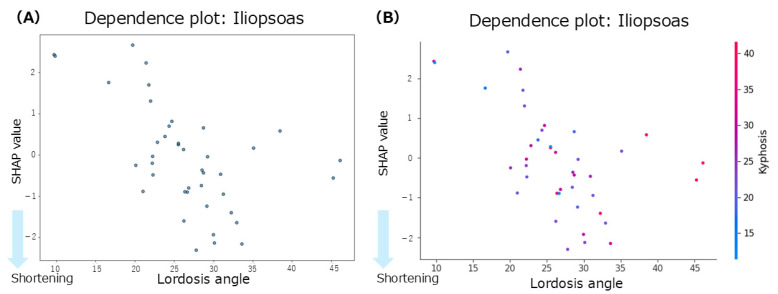
Dependence plots for the iliopsoas muscle. (**A**): dependence plot for the lumbar lordosis angle; (**B**): dependence plot for the interaction between the thoracic kyphosis and lumbar lordosis angles.

The summary plot for the iliopsoas (Figure 3A) shows that greater anterior pelvic tilt angles were associated with negative SHAP values, indicating a contribution to iliopsoas muscle shortening. Notably, even in patients with smaller anterior pelvic tilt angles, negative SHAP values were observed, indicating a shortening tendency. Similarly, increased lumbar lordosis angles were associated with negative SHAP values, suggesting a contribution to the shortening of the iliopsoas. In contrast, the thoracic kyphosis angles showed a mixed distribution of positive and negative SHAP values with no consistent trend. According to the mean SHAP values in Table 2, lumbar lordosis had the strongest individual contribution to iliopsoas muscle shortening (mean SHAP = −0.09). Given that lumbar lordosis was the most influential factor, a dependence plot of the lordosis angle was generated (Figure 4A). This plot revealed that lumbar lordosis angles between 20° and 35° were associated with negative SHAP values, indicating a contribution to iliopsoas shortening.

Focusing on interregional spinal interactions, the mean interaction SHAP values (Table 2) were positive across all spinal region combinations, suggesting that interaction effects did not contribute to iliopsoas shortening. However, a dependence plot of the interaction between lumbar lordosis and thoracic kyphosis (Figure 4B) showed that lumbar lordosis angles between 20° and 40° and thoracic kyphosis angles between 20° and 50° were associated with negative SHAP values, indicating a potential interaction-related contribution to the shortening of the iliopsoas within this specific range.

#### 3.2.2. Rectus Femoris

**Figure 5:**
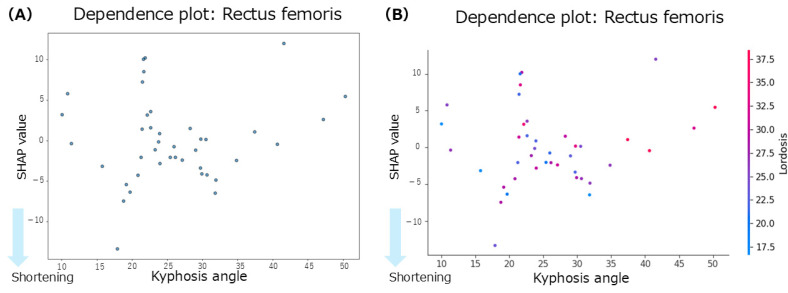
Dependence plots for the rectus femoris muscle. (**A**): dependence plot for the thoracic kyphosis angle. (**B**): dependence plot for the interaction between the thoracic kyphosis and lumbar lordosis angles.

The summary plot for the rectus femoris (Figure 3B) shows that the SHAP values for thoracic kyphosis were positively and negatively distributed with no clear trend observed. However, when the lumbar lordosis angles were high, the SHAP values tended to be negative, indicating a contribution to rectus femoris shortening. Similarly, greater anterior pelvic tilt angles were associated with negative SHAP values, suggesting a tendency towards shortening. According to the mean SHAP values in Table 2, thoracic kyphosis showed the strongest individual contribution to rectus femoris shortening (mean SHAP = −0.05). The dependence plot for thoracic kyphosis (Figure 5A) revealed that the SHAP values were negative within the 10–35° range, indicating a contribution to rectus femoris shortening in this angle range.

Focusing on the interactions between spinal regions, the interaction of SHAP values (Table 2) indicated that the interaction between thoracic kyphosis and lumbar lordosis contributed to rectus femoris shortening (interaction mean SHAP = −0.08). The dependence plot for this interaction (Figure 5B) showed that the SHAP values were negative when the thoracic kyphosis and lumbar lordosis angles were within the 10–35° range, indicating a shortening-related contribution from their interaction.

#### 3.2.3. Hamstrings

**Figure 6:**
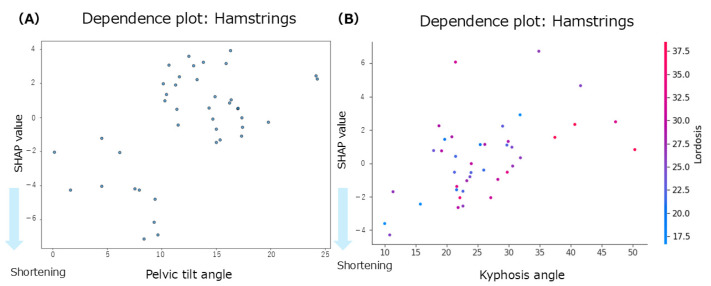
Dependence plots for the hamstrings. (**A**): dependence plot for the anterior pelvic tilt angle; (**B**): dependence plot for the interaction between the thoracic kyphosis and lumbar lordosis angles.

The summary plot for the hamstrings (Figure 3C) shows that greater lumbar lordosis angles were associated with negative SHAP values, indicating a contribution to hamstring shortening. Similarly, smaller anterior pelvic tilt angles were associated with negative SHAP values, suggesting a tendency toward muscle shortening. In contrast, the SHAP values for thoracic kyphosis were positively and negatively distributed with no consistent trend observed. According to the Mean SHAP values in Table 2, pelvic tilt showed the strongest individual contribution to hamstring shortening (Mean SHAP = −0.30). The dependence plot for anterior pelvic tilt (Figure 6A) indicated that the SHAP values were negative in the 0–10° range, suggesting a contribution to hamstring shortening in this angle range.

Focusing on inter-regional spinal interactions, the interaction mean SHAP values (Table 2) showed that both the thoracic–lumbar interaction (interaction mean SHAP = −0.27) and the thoracic–pelvic interaction (interaction mean SHAP = −0.03) contributed to hamstring shortening. The dependence plot for the interaction between thoracic kyphosis and lumbar lordosis (Figure 6B) demonstrated that when thoracic kyphosis was between 10° and 30° and lumbar lordosis was between 18° and 35°, the SHAP values were negative, indicating a contribution to hamstring shortening in this range.

#### 3.2.4. Gluteus Maximus

**Figure 7:**
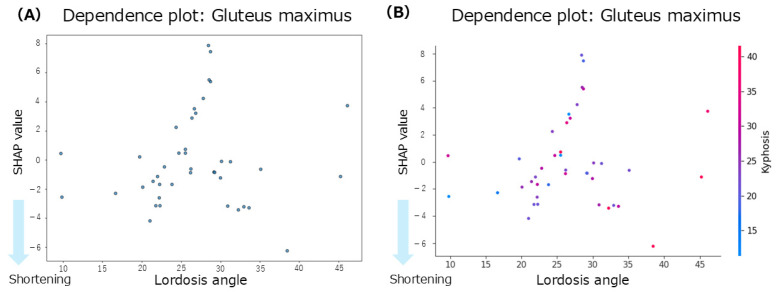
Dependence plots for the gluteus maximus concerning lumbar lordosis. (**A**): Dependence plot for the lumbar lordosis angle. (**B**): Dependence plot for the interaction between the lumbar lordosis and thoracic kyphosis angles.

The summary plot for the gluteus maximus (Figure 3D) showed that the SHAP values for anterior pelvic tilt were positively and negatively distributed, indicating no consistent trend. When the lumbar lordosis angles were small, the SHAP values tended to be negative, suggesting a contribution to shortening of the GM. In contrast, at higher lumbar lordosis angles, the SHAP values were mixed, with no clear directional patterns. High thoracic kyphosis angles were associated with negative SHAP values, indicating their contribution to gluteus maximus shortening. According to the mean SHAP values in Table 2, lumbar lordosis had the strongest individual contribution to gluteus maximus shortening (mean SHAP = −0.08). The dependence plot for lumbar lordosis (Figure 7A) revealed that the SHAP values were negative in the 10–45° range, suggesting a shortening tendency in this range.

Regarding inter-regional spinal interactions, the mean interaction SHAP values (Table 2) showed that the interaction between thoracic kyphosis and lumbar lordosis contributed to gluteus maximus shortening (interaction mean SHAP = −0.16). The dependence plot for this interaction (Figure 7B) indicated that when thoracic kyphosis was between 10° and 40° and lumbar lordosis was between 10° and 45°, the SHAP values were negative, suggesting a contribution to gluteus maximus shortening in this range.

#### 3.2.5. Back Muscles

**Figure 8:**
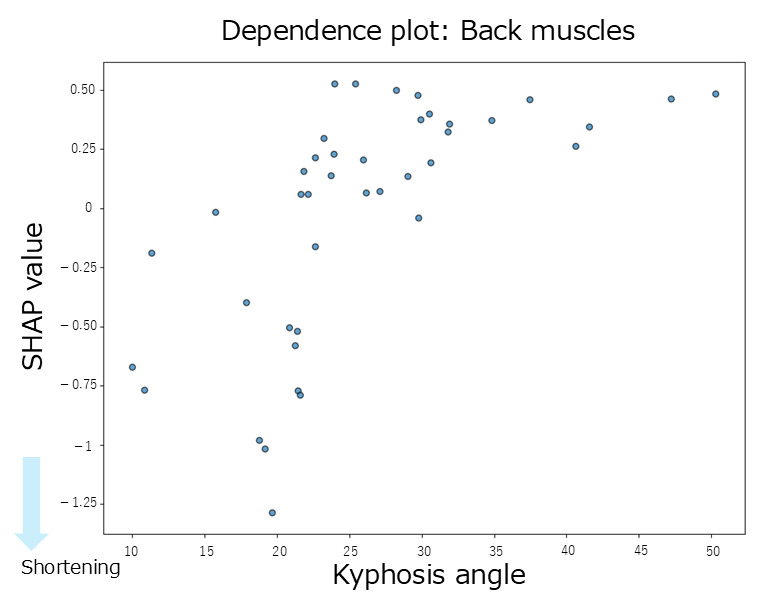
A dependence plot for the back extensor muscles about the thoracic kyphosis angle.

The summary plot for the back extensor muscles (Figure 3E) showed a tendency toward negative SHAP values when the thoracic kyphosis angles were small, indicating a contribution to muscle shortening. In contrast, the SHAP values for pelvic tilt were positively and negatively distributed, showing no clear trends. When the thoracic kyphosis angles were high, the SHAP values tended to be negative, suggesting a shortening effect. According to the mean SHAP values in Table 2, both thoracic kyphosis (mean SHAP = −0.02) and pelvic tilt (mean SHAP = −0.02) contributed to back extensor shortening. The dependence plot for thoracic kyphosis (Figure 8) showed that the SHAP values were negative in the 10–23° range, indicating an association with muscle shortening in that angular range.

Regarding inter-regional spinal interactions, the interaction of SHAP values (Table 2) did not contribute to the shortening of the back extensor muscles. Additionally, the absolute SHAP values for the back extensor muscles were lower than those observed for the hip-related muscles, indicating that both the individual and interaction effects of spinal alignment contributed minimally to the variation in back extensor muscle length.

#### 3.2.6. Abdominal Muscles

**Figure 9:**
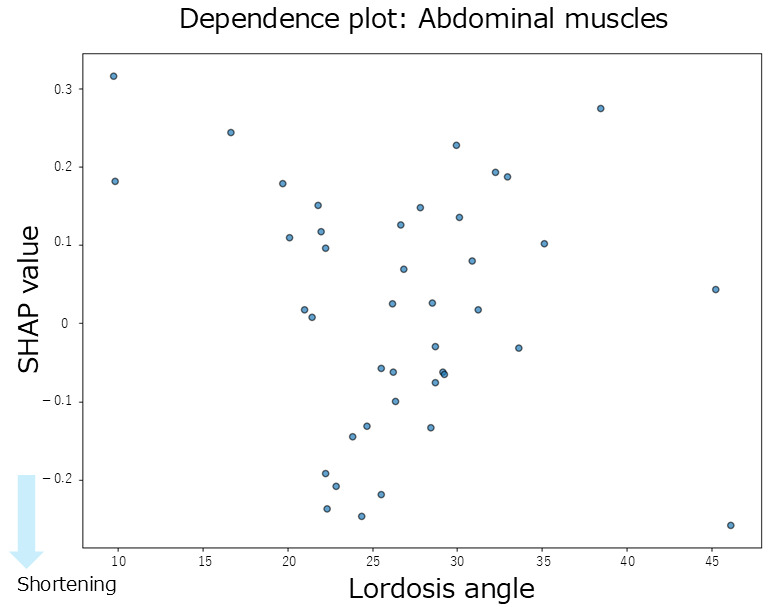
Dependence plot for the abdominal muscles about lumbar lordosis.

The summary plot for the abdominal muscles (Figure 3F) shows that greater thoracic kyphosis angles tended to be associated with positive SHAP values, indicating a contribution to abdominal muscle shortening. Similarly, smaller lumbar lordosis angles also tended to produce positive SHAP values, which contributed to shortening. In contrast, overall, the SHAP values for lumbar lordosis were distributed positively and negatively, showing no consistent trend. According to the mean SHAP values shown in Table 2, lumbar lordosis had the strongest individual contribution to abdominal muscle shortening (mean SHAP = 0.02). The dependence plot for lumbar lordosis (Figure 9) revealed that the SHAP values were positive in the 10–35° range, indicating a shortening-related effect in this region.

Regarding inter-regional spinal interactions, none of the interaction SHAP values between the spinal regions contributed to abdominal muscle shortening (Table 2). Moreover, the absolute SHAP values for the abdominal muscles were lower than those for the hip-related muscles, suggesting that both the individual and interaction effects of spinal alignment had a limited influence on abdominal muscle length.

## 4. Discussion

This study investigated the associations between the individual and interactive effects of thoracic, lumbar, and pelvic alignment and trunk and hip-related muscle lengths. The SHAP summary plots derived from the random forest model revealed that the degree of the spinal alignment’s contribution to muscle shortening varied across different muscles. For the rectus femoris, thoracic alignment contributed the most to muscle shortening, with the interaction between the thoracic and lumbar regions showing the most substantial contribution among the interaction effects. The lumbar alignment had the greatest influence on the iliopsoas; however, no significant interaction effect was observed. Hamstring shortening was associated with pelvic alignment alone and with the interaction between the thoracic and lumbar regions. In the gluteus maximus, the lumbar alignment and thoracolumbar interactions contribute to muscle shortening. In contrast, the contributions from both the individual and interaction effects were minimal for the back extensor and abdominal muscles. These findings suggest that muscle shortening, particularly in the muscles surrounding the hip joint, may be associated with both the individual influence of spinal alignment and interactions between spinal regions.

Regarding the effect of individual spinal alignment on muscle shortening, thoracic kyphosis contributed to the shortening of the rectus femoris, with a mean SHAP value of −0.05. Although the summary plot indicated that thoracic kyphosis had the most significant impact on the rectus femoris, SHAP values exhibited bidirectional contributions (both positive and negative), suggesting no consistent trend. However, the dependence plot revealed that the SHAP values were predominantly negative within the thoracic kyphosis angle range of 10–35°, indicating an association with rectus femoris shortening. According to previous studies, the average thoracic kyphosis angle in healthy young men is 31.2 ± 5.32° [18], and an excessive thoracic kyphosis is defined as an angle of 42° or greater [19]. In the present study, the participants had an average thoracic kyphosis angle of 25.9 ± 8.7°, suggesting a tendency toward smaller than average thoracic curvature. Furthermore, the dependence plot showed a concentration of negative SHAP values, particularly within the 20–30° range, indicating that individuals with reduced thoracic curvature tended to shorten the rectus femoris. Although the rectus femoris originates from the anterior inferior iliac spine and does not attach to the thoracic spine, it is directly connected to the pelvis. Therefore, anterior pelvic tilt places the rectus femoris in a shortened position. This suggests that changes in pelvic alignment may indirectly influence other regions, such as the thoracic kyphosis, through kinetic chains and compensatory mechanisms. Additionally, previous reports have indicated that shortening of the rectus femoris may promote anterior pelvic tilt and increase lumbar lordosis as a compensatory response [20]. This is consistent with the summary plot of the present study, which showed a tendency for rectus femoris shortening with a more significant anterior pelvic tilt, aligning with the anatomical and biomechanical functions of the muscle. These findings suggest that rectus femoris shortening may be associated with changes in pelvic alignment and indirectly with thoracic kyphosis, potentially serving as a compensatory mechanism for maintaining the overall spinal alignment. Moreover, a previous study reported a significant negative correlation between thoracic kyphosis and rectus femoris length, indicating that more significant thoracic kyphosis is associated with rectus femoris shortening [21]. This suggests that, as the thoracic spine becomes more kyphotic and the center of gravity shifts, changes in the rectus femoris length may occur, stabilizing the center of mass over the lumbar spine and pelvis. In summary, the contribution of thoracic kyphosis to the rectus femoris shortening may be interpreted as a compensatory adjustment in the spinal curvature among individuals with average or below-average thoracic kyphosis in response to changes in pelvic and lumbar alignment, although thoracic kyphosis itself does not directly influence the rectus femoris through movement.

Concerning lumbar lordosis, the most significant contributions to muscle shortening were observed for the iliopsoas (mean SHAP = −0.09) and the gluteus maximus (mean SHAP = −0.08). In the summary plot for the iliopsoas, the anterior pelvic tilt showed the highest contribution; however, the SHAP values were widely dispersed, indicating a lack of consistency. In contrast, lumbar lordosis angles within the 20–35° range were associated with negative SHAP values, suggesting a contribution to iliopsoas shortening. Previous research has reported an average lumbar lordosis angle of 30.2 ± 5.21° in healthy young men [18]. In contrast, the average value in the present study was 26.7 ± 7.2°, indicating a tendency toward reduced lumbar curvature in the current sample. The dependence plot further supported this finding, showing a predominance of negative SHAP values in the 25–35° range, indicating an overall trend of iliopsoas shortening. Among the iliopsoas components, the psoas major is directly attached to the lumbar spine and facilitates lumbar lordosis. A previous study reported that shortening of the iliopsoas muscle was associated with increased lumbar lordosis [4]. For the gluteus maximus, the mean SHAP value was −0.08, indicating a contribution to muscle shortening. Although anterior pelvic tilt showed the highest contribution in the summary plot, the SHAP values for this variable were distributed across both positive and negative directions, with no clear trend. The dependence plot revealed negative SHAP values in individuals with lordosis angles > 30° and reduced angles of <30°. Notably, negative SHAP values in participants with reduced lumbar lordosis support previous findings, suggesting that a smaller lumbar lordosis angle contributes to gluteus maximus shortening [22]. The gluteus maximus attaches to the posterior aspect of the pelvis and plays a role in posterior pelvic tilt. In a posture in which the posterior pelvic tilt reduces lumbar lordosis, gluteus maximus shortening may occur. A previous study reported that individuals with gluteus maximus contracture demonstrated decreased lumbar lordosis compared with healthy individuals [23]. This has been interpreted as a compensatory mechanism for maintaining the global spinopelvic balance through pelvic morphological changes. Although some variability exists in the lumbar lordosis angle at which gluteus maximus shortening occurs, the finding that reduced lumbar lordosis is associated with gluteus maximus shortening is consistent with those of previous studies [4]. These results suggest that specific ranges of lumbar lordosis angles contribute to the shortening of the iliopsoas and gluteus maximus. This may be explained by a combination of direct anatomical effects and indirect compensatory mechanisms involving other body segments, highlighting the multifactorial relationship between lumbar alignment and muscle length.

Regarding anterior pelvic tilt, the hamstrings showed the most substantial contribution to muscle shortening, with a mean SHAP value of −0.30. The summary plot also indicated that smaller anterior pelvic tilt angles were associated with hamstring shortening, suggesting a tendency towards muscle shortening in the presence of a posterior pelvic tilt. The dependence plot further confirmed this relationship, with many participants exhibiting negative SHAP values when the anterior pelvic tilt was ≤10°, indicating a contribution to muscle shortening. In this study, the average anterior pelvic tilt angle was 12.6 ± 5.1°, which is consistent with previous reports indicating an average of 12.6 ± 5.8° in healthy young men [13]. According to a previous study [13], the primary role of the hamstrings in standing posture is to induce posterior pelvic tilt. As hip extensors, the hamstrings pull the posterior pelvis downward, counteracting an excessive anterior tilt. Similarly, a previous study reported that the hamstrings promote posterior pelvic tilt [4]. Moreover, a previous study noted that hamstring shortening is generally associated with a significant increase in posterior pelvic tilt, suggesting a relationship between reduced hamstring flexibility and increased pelvic retroversion [24]. However, in the present study, some participants who did not show a posterior pelvic tilt still exhibited SHAP values, indicating hamstring shortening. A previous study reported that hamstring length is not always associated with anterior pelvic tilt during standing [25]. They suggested that when multiple muscle groups are simultaneously shortened, their effects may cancel each other out, resulting in no observable alignment changes. Nevertheless, in our study, the contribution of hamstring shortening was relatively small in participants without a posterior pelvic tilt, whereas it was substantially greater in those with a posterior pelvic tilt. These findings suggest that, when a posterior pelvic tilt is present, the hamstrings, which attach directly to the pelvis, may have direct anatomical and biomechanical roles in muscle shortening.

This study examined whether interactions between spinal regions enhance muscle shortening. Using interaction SHAP analysis, we found that all hip-related muscles contributed the most to muscle shortening through the interaction between thoracic kyphosis and lumbar lordosis. In contrast, for the back extensors and abdominal muscles, the contributions from both individual and interaction effects were minimal. When comparing the contributions of individual versus interactive effects, thoracic kyphosis alone contributed to rectus femoris shortening; however, the thoracolumbar interaction showed an even more significant contribution. Similarly, thoracolumbar interaction influenced gluteus maximus shortening more strongly than lumbar lordosis alone. For the hamstrings, pelvic alignment showed the strongest individual contribution, but interaction SHAP analysis revealed that the thoracolumbar interaction was the dominant factor. In contrast, lumbar lordosis showed the strongest individual contribution to the iliopsoas; however, the interaction effects were weaker. Based on these findings, we further explored the dependence plots for the rectus femoris, gluteus maximus, and hamstrings, which showed increased contributions under interaction effects, to identify the characteristic angle ranges. For the rectus femoris, participants with thoracic kyphosis angles of 20–30° and lumbar lordosis angles of 20–35° exhibited concentrated negative SHAP values, indicating a contribution to muscle shortening. For the gluteus maximus, a similar concentration of negative SHAP values was observed in the ranges of thoracic kyphosis 20–30° and lumbar lordosis 15–30°. Similarly, for the hamstrings, negative SHAP values were concentrated in the range of thoracic kyphosis 10–30° and lumbar lordosis 18–35°. Despite their different anatomical locations and functions (i.e., anterior versus posterior hip musculature), these muscles share similar thoracolumbar interaction ranges associated with shortening. A previous study suggested that although the rectus femoris does not anatomically attach to the thoracic spine, thoracic kyphosis may still indirectly influence its function via compensatory adjustments of the spine and pelvis to maintain postural balance [21]. Similarly, a previous study reported that individuals with gluteus maximus contracture showed reduced lumbar lordosis and increased thoracic kyphosis, indicating that compensatory mechanisms in spinopelvic alignment may preserve the overall sagittal balance even in symptomatic populations [23]. For the hamstrings, increased tension has been reported to influence spinal curvature [26], and associations between lumbar lordosis and anterior pelvic tilt in the standing position have also been established [27], suggesting that spinal and pelvic alignments jointly contribute to postural regulation. Physiological spinal curvatures, such as lordosis and kyphosis, help efficiently absorb and distribute mechanical loads. These curvatures also influence the position of the center of gravity, thereby contributing to balance and postural stability. Curvature levels, angles, and combinations of spinal and pelvic alignments vary widely and can be classified into multiple alignment types [10]. Taken together, these findings suggest that the spine and pelvis function as a coordinated complex, the spinopelvic unit, which adjusts posture in response to changes in the center of gravity and load distribution. Therefore, changes in thoracic kyphosis and lumbar lordosis may influence pelvic alignment through kinetic chains, and the interaction between these regions may play a key role in contributing to the shortening of the hip-related muscles.

The novelty and strength of this study lie in its investigation of how individual spinal alignments and inter-regional spinal interactions are associated with muscle length, using a machine learning-based analytical method that enables interpretability. The analysis revealed that among the hip-related muscles, individual spinal regions tended to contribute to muscle shortening. Furthermore, in the rectus femoris, hamstring, and gluteus maximus, the combined effects of lumbar lordosis and thoracic kyphosis were associated with a more substantial contribution to muscle shortening. These findings suggest that the direct anatomical effects of muscles attached to the spine and indirect effects, such as spinal curvature-induced shifts in the center of gravity and compensatory adjustments due to muscle length imbalance, play a role in whole-body postural balance. The results of this study enhance the interpretation of the relationship between standing postural alignment and muscle shortening, offering the potential for more integrated and precise interpretations of combined postural and muscle assessments. Moreover, if muscle-shortening tendencies in spinal alignment can be understood, it is possible to prioritize intervention strategies targeting specific muscles based on their relevance to postural alignment. Consequently, the interpretative framework established in this study regarding the relationship between spinal alignment and muscle length may contribute to improved clinical assessments and more effective treatment decision-making.

The present study has several limitations. This study focused on the individual associations between spinal alignment and the lengths of specific muscles. As such, it did not account for the interactive influence of the agonist and antagonist muscle groups. Given the variability in muscle development and usage patterns influenced by individual motor experiences and neuromuscular adaptations, antagonistic muscle activity may influence spinal alignment and muscle lengths. The study sample consisted exclusively of young adult males, which limits the generalizability of the findings to other populations, including females and older adults. This restriction intentionally excludes the confounding effects of sex and spinal pathology. Future studies should examine whether the relationships identified in this study differ by sex or age, as muscle characteristics and spinal alignment may vary accordingly. The analysis in this study was limited to the sagittal plane alignment. Frontal plane factors such as scoliosis were not assessed, and the precise vertical level of the spinal curvatures (e.g., curvature apex) was not examined in detail. Previous research has indicated that spinal alignment varies significantly depending on the combination of thoracic, lumbar, and pelvic configurations [10]. Future studies should include frontal plane alignment and curvature apex positioning to examine detailed and characteristic relationships between spinal posture and muscle shortening. While this study focused on muscle length, it is important to recognize that muscle strength influences spinal alignment. The muscle strength data were not collected in this study. While we focused on muscle length indices estimated from postural data, the potential influence of muscle strength on postural alignment and compensatory mechanisms remains unexplored. Future research should incorporate strength measurements to more comprehensively understand the interaction between spinal alignment, muscle function, and flexibility. Therefore, the results cannot be interpreted as a comprehensive analysis of all the spinal alignment factors. Future investigations should incorporate a broader range of variables, including strength and connective tissue properties, to further clarify the contribution of muscle length to spinal alignment within a multidimensional framework. These findings may have clinical implications in rehabilitation strategies; for instance, targeted flexibility exercises for the rectus femoris could potentially reduce compensatory increases in thoracic kyphosis, thereby improving sagittal balance in individuals with anterior pelvic tilt. Future research directions may also be highlighted.

## 5. Conclusions

This study investigated how the individual effects and interregional interactions of thoracic, lumbar, and pelvic alignments are associated with muscle length. These findings suggest that individual spinal alignment and the interaction between multiple spinal regions contribute to muscle shortening, particularly in the hip-related musculature. Among these, the interaction between thoracic kyphosis and lumbar lordosis showed the strongest association with muscle shortening, indicating that the combined influence of thoracic and lumbar alignments may play a key role in shortening hip-related muscles.

## Figures and Tables

**Table 1:** Characteristics of spinal alignment and muscle length indices in study participants.

Measurement Index	Value (Mean ± SD) (*n* = 40)
Thoracic kyphosis angle (°)	25.9 ± 8.7
Lumbar lordosis angle (°)	26.7 ± 7.2
Anterior pelvic tilt angle (°)	12.6 ± 5.1
Iliopsoas (°)	−9.8 ± 5.2
Rectus femoris (°)	70.8 ± 14.1
Hamstrings (°)	140.4 ± 10.0
Gluteus maximus (°)	69.0 ± 10.8
Back extensor muscles (cm)	21.2 ± 1.1
Abdominal muscles (cm)	12.3 ± 0.7

**Table 2:** Mean SHAP values for individual and interaction effects of spinal alignment on each muscle length index.

	RF	IP	HAM	GM	BA	AB
Kyphosis	−0.05	0.00	0.29	0.05	−0.02	−0.01
Lordosis	0.16	−0.09	0.16	−0.08	0.00	0.02
Pelvic	0.02	0.07	−0.30	0.27	−0.02	−0.01
Kyphosis × Lordosis	−0.08	0.00	−0.27	−0.16	0.00	0.00
Kyphosis × Pelvic	−0.05	0.01	−0.03	−0.04	0.00	0.00
Lordosis × Pelvic	0.00	0.04	0.06	0.19	−0.01	−0.01
Kyphosis	−0.05	0.00	0.29	0.05	−0.02	−0.01

RF: rectus femoris, IP: iliopsoas, HAM: hamstrings, GM: gluteus maximus, BA: back muscles, AB: abdominal muscles.

## Data Availability

The original contributions presented in this study are included in the article. Further inquiries can be directed to the corresponding authors.
